# HIV viremia is associated with compromised SARS-CoV-2 Beta variant neutralization

**DOI:** 10.1093/infdis/jiac343

**Published:** 2022-08-17

**Authors:** Shi-Hsia Hwa, Jumari Snyman, Mallory Bernstein, Yashica Ganga, Sandile Cele, Daniel Muema, Chee Wah Tan, Khadija Khan, Farina Karim, Willem Hanekom, Leslie Bernstein, Stefan H E Kaufmann, Lin-Fa Wang, Thumbi Ndung’u, Alex Sigal

**Affiliations:** Africa Health Research Institute, Durban, South Africa; Division of Infection and Immunity, University College London, London, United Kingdom; Africa Health Research Institute, Durban, South Africa; HIV Pathogenesis Programme, University of KwaZulu-Natal, Durban, South Africa; School of Laboratory Medicine and Medical Sciences, University of KwaZulu-Natal, Durban, South Africa; Africa Health Research Institute, Durban, South Africa; Africa Health Research Institute, Durban, South Africa; Africa Health Research Institute, Durban, South Africa; School of Laboratory Medicine and Medical Sciences, University of KwaZulu-Natal, Durban, South Africa; Africa Health Research Institute, Durban, South Africa; HIV Pathogenesis Programme, University of KwaZulu-Natal, Durban, South Africa; School of Laboratory Medicine and Medical Sciences, University of KwaZulu-Natal, Durban, South Africa; Programme in Emerging Infectious Diseases, Duke-NUS Medical School, Singapore, Singapore; Africa Health Research Institute, Durban, South Africa; School of Laboratory Medicine and Medical Sciences, University of KwaZulu-Natal, Durban, South Africa; Africa Health Research Institute, Durban, South Africa; School of Laboratory Medicine and Medical Sciences, University of KwaZulu-Natal, Durban, South Africa; Africa Health Research Institute, Durban, South Africa; Division of Infection and Immunity, University College London, London, United Kingdom; Department of Population Sciences, Beckman Research Institute, City of Hope Comprehensive Cancer Center, Duarte, CA, USA; Max Planck Institute for Infection Biology, Berlin, Germany; Max Planck Institute for Multidisciplinary Sciences, Göttingen, Germany; Hagler Institute for Advanced Study, Texas A&M University, College Station, TX, United States; Programme in Emerging Infectious Diseases, Duke-NUS Medical School, Singapore, Singapore; SingHealth Duke-NUS Global Health Institute, Singapore, Singapore; Africa Health Research Institute, Durban, South Africa; Division of Infection and Immunity, University College London, London, United Kingdom; HIV Pathogenesis Programme, University of KwaZulu-Natal, Durban, South Africa; School of Laboratory Medicine and Medical Sciences, University of KwaZulu-Natal, Durban, South Africa; Ragon Institute of Massachusetts General Hospital, Massachusetts Institute of Technology and Harvard University, Cambridge, MA, USA; Africa Health Research Institute, Durban, South Africa; School of Laboratory Medicine and Medical Sciences, University of KwaZulu-Natal, Durban, South Africa; Max Planck Institute for Infection Biology, Berlin, Germany; Centre for the AIDS Programme of Research in South Africa, Durban, South Africa

**Keywords:** SARS-CoV-2, COVID-19, Beta variant, HIV, antiretroviral therapy, antibodies, neutralization

## Abstract

**Background:**

SARS-CoV-2 infection may be associated with worse clinical outcomes in people with HIV (PWH). We report anti-SARS-CoV-2 antibody responses in COVID-19 hospitalized patients in Durban, South Africa during the second SARS-CoV-2 infection wave dominated by the Beta (B.1.351) variant.

**Methods:**

Thirty-four participants with confirmed SARS-CoV-2 infection were followed up with weekly blood sampling to examine antibody levels and neutralization potency against SARS-CoV-2 variants. Participants included 18 PWH, of whom 11 were HIV viremic.

**Results:**

SARS-CoV-2 specific antibody concentrations were generally lower in viremic PWH relative to virologically suppressed PWH and HIV-negative participants and neutralization of the Beta variant was 4.9-fold lower in viremic PWH. Most HIV-negative participants and ART-suppressed PWH also neutralized the Delta (B.1.617.2) variant, whereas the majority of viremic PWH did not. CD4 counts <500 cells/μL were associated with lower frequencies of IgG and IgA seroconversion. In addition, there was a high correlation between a surrogate virus neutralization test and live virus neutralization against ancestral SARS-CoV-2 virus in both PWH and HIV-negative individuals, but correlation decreased for the Beta variant neutralization in PWH.

**Conclusions:**

HIV viremia was associated with reduced Beta variant neutralization. This highlights the importance of HIV suppression in maintaining an effective SARS-CoV-2 neutralization response.

## Background

The second epidemic wave of COVID-19 in South Africa was dominated by the Beta variant of concern (20H/501Y.V2, Pango lineage B.1.351) which emerged in the Eastern Cape Province. By mid-November 2020, Beta represented the majority of sequenced samples [1]. Spike mutations in the receptor-binding domain (RBD) and N-terminal domain result in partial antigenic escape of Beta from neutralizing antibody immunity elicited by ancestral strains [2, 3], and the efficacy of the ChAdOx1 nCoV-19 vaccine in preventing mild-to-moderate COVID-19 dropped from 75% before 31 October 2021, to 10% when the Beta variant became prevalent [4].

People with HIV (PWH) may be at higher risk for COVID-19 mortality [5] and for more severe COVID-19 outcomes, [6-8]. This may be due to an impaired T cell and antibody response to SARS-CoV-2 infection in PWH [9], as neutralizing antibodies are correlated with vaccine efficacy and protection against COVID-19 [10]. We found no differences in the antibody responses of COVID-19 PWH versus HIV negative participants in the first infection wave in South Africa prior to the emergence of variants of concern [11]. However, we observed higher disease severity in PWH in our cohort of infected, unvaccinated participants during the Beta (but not the ancestral virus) infection wave [6]. Therefore, we re-examined antibody neutralizing immunity in PWH in the Beta infection wave.

Here we evaluated whether, during the second infection wave dominated by the Beta variant, PWH differed in their infection elicited antibody responses to SARS-CoV-2. We measured isotype-specific spike RBD-binding and virus neutralizing antibody responses within the first 60 days post-Covid-19 diagnosis in PWH and HIV-negative COVID-19 participants. We also evaluated the suitability of a commercial surrogate virus neutralization test (sVNT) in this patient population [12]. In agreement with our previous report showing more severe COVID-19 infection outcomes and altering of immune responses in PWH in the Beta-dominated second infection wave in South Africa [6] and lower levels of Delta (B.1.617.2) neutralization capacity in unvaccinated PWH [13], we observed lower Beta-infection elicited neutralization capacity of the Beta variant in PWH with detectable HIV viremia.

## Methods

### Ethical statement and study participants

The study location and sampling methodology have been previously described [6]. The study protocol was approved by the University of KwaZulu-Natal Biomedical Research Ethics Committee (reference number BREC/00001275/2020). Written informed consent was obtained for all enrolled participants. Hospitalized patients with SARS-CoV-2 infection in Durban, KwaZulu-Natal, South Africa were enrolled in the study and followed up weekly with collection of oropharyngeal/nasopharyngeal swab and whole blood samples at each study visit. Inclusion criteria were SARS-CoV-2 infection confirmed by reverse transcription-quantitative polymerase chain reaction (RT-qPCR) and age over 18 years. All participants meeting inclusion criteria were eligible for enrolment.

For analyses of antibody responses, we selected participants who had been enrolled during the second, Beta dominated COVID-19 infection wave in South Africa and had a baseline blood sample at enrolment and one or more additional samples covering the first month post-diagnostic swab (dates of diagnosis ranged from 30 December 2020 – 01 April 2021). None of the participants were vaccinated at the time of collection. Since date of symptom onset depended on recall, which may vary across participants, we used days post-diagnostic swab (DPD) for longitudinal analyses. Eighteen PWH were available within that time period and we arbitrarily selected the first 16 HIV negative participants who also fit these criteria in order to have a similar number of controls. COVID-19 vaccines had not yet been made available to the general population in South Africa during the study period and none of the participants had been vaccinated during the sampling period included in these analyses.

### Laboratory testing

RT-qPCR for SARS-CoV-2 ORF1ab, S, and N genes was performed. Commercial diagnostic laboratories performed testing for HIV viral load (Molecular Diagnostic Services, Durban, South Africa) and CD4 and CD8 cell counts (Ampath, Durban, South Africa). We defined viremia as any viral load above the limit of detection of 40 copies/mL. The presence of antiretroviral therapy (ART) components in plasma of PWH was measured by LC-MS/MS [6].

### ELISA

Isotype-specific RBD enzyme-linked immunosorbent assays (ELISAs) were performed as previously described [11]. Briefly, plates were coated with recombinant SARS-CoV-2 receptor-binding domain (gift from Galit Alter), blocked, and incubated with plasma sample dilutions. Secondary (detection) antibodies for IgG, IgM, and IgA were isotype-specific, cross-adsorbed, horseradish-peroxidase (HRP)-conjugated polyclonal antibodies. For each isotype, an RBD-binding monoclonal antibody was used to generate a standard curve for interpolating concentrations of anti-RBD specific antibodies, namely CR3022 IgG (gift from Galit Alter), hIgM2001 (GenScript), and hIgA2001 (GenScript). Pre-pandemic plasma from HIV-uninfected individuals and commercial human serum (EU/USA origin, BioWest) were used to establish baselines per isotype as previously described [11].

### Surrogate virus neutralization test (sVNT)

A surrogate virus neutralization test based on detecting inhibition of recombinant human angiotensin-converting enzyme 2 (ACE2) binding to RBD:peroxidase fusion protein was performed according to the manufacturer's instructions (GenScript SARS-CoV-2 Surrogate Virus Neutralization Test, version RUO 3.0). All samples were tested at a single dilution of 1:10. Sample results are reported as percent inhibition relative to the kit negative control, with a manufacturer-recommended positive cut-off value of ≥30%.

### Cells

Vero E6 cells (ATCC CRL-1586) were obtained from Cellonex in South Africa and propagated as previously described. An in-house cell line, H1299-ACE2, was generated by infecting H1299 (ATCC CRL-5803) with an ACE2-overexpressing stable lentiviral vector [3].

### Viruses

SARS-CoV-2 D614G, Beta, and Delta isolates used in these experiments are described in our previous work [3, 14]. Passage 3 stocks were used. All work with live virus was performed in Biosafety Level 3 containment using protocols for SARS-CoV-2 approved by the Africa Health research Institute Biosafety Committee.

### Virus neutralization assay

Authentic virus neutralization assays of plasma antibodies based on reduction of immunostained focus-forming units per well were performed using a similar procedure as in our previous work [3, 13-15] with the following modifications due to the larger size of Beta virus foci: we reduced the input viral load to 70 focus forming units/well and shortened the incubation time to 18 hours post-infection for all three D614G (first wave), Beta, and Delta isolates to minimize overlapping foci. Plates were fixed, stained, scanned, and counted as previously described.

Focus counts per well were normalized against the average of the no-antibody virus control wells on each plate. Fifty percent focus reduction neutralization titers ($FRNT_{50}$) expressed as the inverse of the sample dilution were calculated in Prism by fitting normalized focus counts for each sample to the four-parameter Hill equation, with the bottom and top parameters constrained to a range of 0 to 1. These included extrapolated values for a few samples that had marginally detectable neutralization at the lowest tested dilution of 1:20. Samples with no neutralization at all were assigned a value of 1 (0 $\log_{10}$). A rabbit monoclonal antibody BS-R2B2 (GenScript A02051) was included as a positive control in each run. The $FRNT_{50}$ of BS-R2B2 was 7.4 ng/mL against D614G and 5.0 ng/mL against Beta virus.

### Statistics

Prism (v9, GraphPad) and Stata (v17, StataCorp) were used for data analysis. Standard statistical methods, including $\chi^2$, Fisher's Exact Test, Friedman test, Kruskal-Wallis test, and Mann-Whitney U test were used to compare groups and estimate relationships between variables. To compare sVNT versus neutralization assay, a four-parameter logistic model with bottom and top constrained to 0% – 100% was used since the sVNT readout is percent inhibition relative to assay controls. To examine the effects of clinical factors on antibody seroconversion and loss, we used Mantel-Haenszel methods to determine univariate and multivariate-adjusted rate ratios and corresponding 95% confidence intervals (CI). Results presented here are univariate as sample size was a limitation for multivariate adjustment and multivariate results. P values <0.05 were considered statistically significant and all statistical tests are two-sided.

## Results

Participants in this study had qPCR confirmed SARS-CoV-2 infection during the Beta infection wave in South Africa [14], with dates of diagnosis ranging from end of December 2020 to start of April 2021. No study participants were vaccinated at the time of collection, and to the best of our knowledge immunity measured here resulted from SARS-CoV-2 infection in the second infection wave in South Africa. Samples from 34 participants were analyzed, and these included 18 (53%) PWH and 16 HIV negative participants (Table 1). Seven out of the 18 PWH had a history of TB (p=0.008, Fisher's exact test). Eleven of the 18 PWH were viremic, and 13 had CD4 counts >500 cells/μL upon enrolment. Median CD4 counts were significantly lower in viremic than suppressed PWH (viremic median 161, IQR 9 – 453; suppressed median 713, IQR 191 – 746; p=0.035). Five of 11 (45%) viremic PWH and all PWH with suppressed HIV viremia had detectable ART at enrolment. Disease severity was higher in the PLW group, but this difference was not statistically significant.

We monitored changes in SARS-CoV-2 spike RBD antibody levels weekly up until about 1-month post-enrolment, where enrolment was visit 1, and there were 5 visits over the one-month period. An additional collection was performed after the one-month period if the participant was available (visit 6). Collection points per participant are graphed in Figure 1. The majority of the 34 participants in this analysis provided samples at weekly follow-up visits 2 through 5 (25, 25, 27, and 31 participants respectively) and 13 participants at visit 6. For IgG (Figure 1A), IgA (Figure 1B), and the surrogate virus neutralization test (Figure 1C), viremic PWH antibodies trended lower compared to HIV-negative and suppressed PWH. IgG and sVNT responses were already above assay cutoff values (1160 ng/mL IgG; 30% sVNT) at the earliest timepoint sampled for the majority of HIV-negative (69% IgG+, 75% sVNT+) and suppressed PWH (71% IgG+, 71% sVNT+), but not for viremic PWH (18% IgG+, 36% sVNT+). Maximum IgG attained during the sampling period was higher in HIV-negative than viremic PWH (p=0.03), but no other significant differences in maximum antibody titers were observed (Figure 1D-F). Proportions of viremic individuals who seroconverted at any point in these three assays were lower than suppressed or HIV-negative individuals (Figure 1G-I); however, these differences were not significant.

Virus neutralization assays were conducted for the closest available sample to 1-month post-diagnosis per participant (median 29 days, IQR 24 – 33) against live virus isolates of D614G (first infection wave/ancestral), Beta (same infection wave), and Delta (following wave). Given that there were no detectable differences between HIV negative and HIV suppressed participants (Figure 1) neutralization of different variants/strains was compared in a combined group of HIV-negative and suppressed PWH (Figure 2A) and in viremic participants (Figure 2B) to increase statistical power. The geometric mean titer (GMT) $FRNT_{50}$ in HIV negative and HIV suppressed participants against D614G virus was 51.7, against Beta virus was 60.9; and against Delta virus was 21.1, slightly above the assay limit of quantification of 1:20 minimum tested dilution (Figure 2A). GMT $FRNT_{50}$ against all three variants of plasma from viremic PWH were below the limit of quantification (Figure 2B). Relative to HIV- and HIV suppressed individuals, viremic participants showed a trend toward lower neutralization of the ancestral strain (Figure 2C), the Beta virus (Figure 2D) and Delta virus (Figure 2E); this was significant for Beta. However, the exact fold-change was difficult to determine since neutralization capacity in multiple viremic participants was below the limit of quantification (LOQ) of a 1:20 plasma dilution. Proportions of viremic PWH who had quantifiable neutralization, defined as titers above LOQ at the 1-month post-diagnosis timepoint, trended lower relative to suppressed PWH and HIV-negative participants for all three variants (Figure 2F-H); this difference had borderline significance for Delta (56% of HIV-negative participants had neutralization above LOQ compared to 86% of suppressed PWH and 27% of viremic PWH; chi-square test p=0.0499).

We investigated associations between participant parameters and antibody levels (Figure S1). Moderate/severe COVID-19, defined as at least requiring supplemental oxygen, was not significantly associated with antibody levels. Age ≥45 years was significantly associated with higher frequencies of IgG and IgA, as well as higher rate ratios based on the Mantel-Haenszel method (RR; IgG 3.8; IgA 4.1). Male participants had a lower rate of IgG seroconversion than females (RR 0.40). $CD4^+$ counts lower than 500 cells/μL were associated with lower frequencies of IgG (Fisher's Exact test p=0.040) and IgA (p=0.025) seroconversion. Lower rates of IgA seroconversion were associated with both HIV viremia (RR 0.35) and $CD4^+$ counts <500 (RR 0.34). Participants with a previous TB diagnosis had higher IgA seroconversion RR of 3.2.

We also compared the surrogate virus neutralization test with the authentic virus neutralization assay, including the first wave plasma samples previously described [11]. Compared to virus neutralization of D614G virus, coefficients of determination were similar for first wave and second wave participants ($R^2$ of 0.88 and 0.88 respectively; Figure 3A, B). Model fit was significantly different between HIV-negative and PWH only for Beta virus neutralization by Beta infection wave samples (p=0.014; Figure 3C). Goodness of fit was lower when comparing the sVNT to Beta virus neutralization ($R^2$=0.61 for PWH, 0.83 for HIV-negative patients; Figure 3C). sVNT false positives compared to virus neutralization were 4/79 (5.1%) for first (ancestral) infection wave samples vs. D614G, 3/95 (3.2%) for second (Beta) infection wave samples vs. D614G, and 1/95 (1.1%) for second wave samples vs. Beta.

Finally, we compared the ELISA, virus neutralization (for D614G), and sVNT results across all first and second wave samples (Figure S2). IgG concentration was most strongly correlated with virus neutralization titers and surrogate neutralization percent inhibition.

## Discussion

We found that HIV viremia attenuates antibody neutralization capacity elicited by Beta variant infection. These results contrast with our findings on participants infected with ancestral SARS-CoV-2 during the first infection wave in South Africa, where no statistically significant differences in antibody responses were found between PWH and HIV-negative participants [11]. However, a much smaller proportion of patients in the first wave study were viremic. We note that the proportion of viremic participants doubled in the second infection wave [6] and may explain the result, although Beta variant specific factors should not be ruled out.

At one-month post-diagnosis, the viremic PWH group, over half of whom showed no detectable ART in the blood, had a lower proportion of neutralizing samples and mean neutralization titers. At the time the samples were collected, vaccines were not yet available to the general population in South Africa (see https://sacoronavirus.co.za/latest-vaccine-statistics/ for vaccine administration over time in South Africa) and the study participants were unvaccinated. Furthermore, re-infection by the Beta variant in the second infection wave of people previously infected with ancestral virus in the South African first infection wave was reported to be rare [16]. Therefore, the effect of HIV viremia on Beta neuralization capacity is measured here in a relatively homogeneous group of participants with likely no previous SARS-CoV-2 immunity.

Against the Delta variant, plasma from HIV-negative participants showed a statistically significant decrease in neutralization relative to earlier variants, similar to previous findings by us and others [14, 17, 18]. The majority of viremic PWH could not neutralize Delta, and therefore may have even lower protection. Impaired CD4 and IgG specific responses to SARS-CoV-2 antigens have also been observed in PWH with active TB [9]. Other arms of the immune system such as CD8 T cells may offer cross-protection from newer variants such as Omicron as most CD8 epitopes in the S protein appear to be conserved in HIV-negative donors [19, 20], however, this remains to be determined in PWH.

In our previous work we showed that impaired neutralization of the Delta variant in COVID-19 convalescent PWH mostly affected persons with sub-optimal HIV suppression [13], consistent with another study [21]. PWH with low/undetectable HIV viral loads vaccinated with ChAdOx1 nCoV-19 adenoviral vectored vaccine or BNT162b2 mRNA vaccine developed robust anti-S and neutralizing antibody responses [4, 22-24]. In contrast, case reports of PWH with advanced HIV disease with low CD4 T cell counts showed reduced antibody responses, delayed SARS-CoV-2 clearance, SARS-CoV-2 evolution of escape mutations and a poor response to vaccination [14, 25-27]. In a cohort study where 11.7% of PWH were viremic, PWH overall had lower anti-RBD IgG and sVNT titers than HIV-negative subjects, although the authors did not stratify by viremia [7].

During the pandemic, several countries including South Africa have reported decreases in HIV testing, ART initiation, or ART collection for various reasons including stress on healthcare systems, lockdowns, and global disruptions to shipping and drug supplies [28]. We have documented lower ART coverage and an increase of the frequency of HIV viremia among COVID-19 patients in the Beta variant infection wave of hospital admitted participants enrolled in our cohort [6]. SARS-CoV-2 spike mutations resembling variants of concern have also been observed to evolved in immunosuppression due to advanced HIV and other immunosuppressed conditions [14, 29].

Finally, we have shown that a surrogate neutralization test which measures blocking of the S RBD-hACE2 interaction correlated well with the live virus neutralization assay in South African convalescent plasma samples including from PWH, although the correlation between sVNT versus virus neutralization in Beta infection wave samples was lower, and this was most pronounced for PWH. The reason for this is unclear. Reasons may include a shift in the binding of neutralizing antibodies away from the RBD in Beta variant infected PWH, which makes the RBD region tested by sVNT less representative of the neutralization response overall. We have previously observed that Beta variant infection leads to an antibody response which is more concentrated on residues 443 to 452 of the spike RBD and less affected by mutations at residue 484 relative to ancestral virus elicited immunity [30]. It is possible that such shifts are not limited to the RBD and may include shifts to other domains such as the spike N-terminal domain [31]. The focus on the RBD may be a limitation of the sVNT approach.

A limitation to the study is the small sample size due to the logistics of sample collection during lockdown due to the Beta epidemic wave in South Africa. Of the total 92 participants enrolled during the second wave study period, only the 34 included in this analysis were available for blood sampling at 1-month post-diagnostic swab. This may have been a result of the strict lockdown which limited mobility post-discharge.

The small sample size in this study may have made the higher COVID-19 disease severity we observed previously between PWH and HIV negative participants [6] to be non-statistically significant. Increased disease severity correlates with higher antibody levels and neutralization capacity [32]. Yet, we measured lower neutralization capacity in viremic PWH. If disease severity is indeed higher in this group of PWH, it may indicate that we are underestimating the interference of HIV viremia with development of neutralization capacity to Beta variant infection. We may also be underestimating the attenuation of neutralization capacity due to suppressed HIV infection, since we detected little difference between HIV suppressed versus HIV negative participants despite possible increased disease severity in the HIV suppressed group.

To conclude, we have found that HIV infection which is not effectively suppressed by ART compromises the neutralizing antibody response to SARS-CoV-2 in the South African population. This shows that the level of HIV suppression, not HIV status alone, may modulate the neutralizing immune response to SARS-CoV-2 variants. ART administration and adherence is key to protecting PWH from adverse outcomes with SARS-CoV-2 infection.

## Acknowledgements

We would like to thank Galit Alter (Ragon Institute) for the receptor-binding domain and CR3022 antibody used in the ELISAs, as well as the draft ELISA protocol which we used as a basis for developing our version. We would like to thank Katya Govender and John Adamson at AHRI for LC-MS/MS analysis.

## Figure legends

**Figure 1: Effect of HIV status and suppression on anti-SARS-CoV-2 RBD antibodies.** Anti-RBD antibody concentrations and surrogate virus neutralization test values (sVNT) in HIV-negative COVID-19 participants (purple), virologically suppressed PWH (green), and viremic PWH (orange). Individual participants' data points are shown. Linear trends on pooled data are shown as transparent ribbons with 95% confidence intervals shown by thick dotted lines. For IgG and IgA, baseline cutoffs indicated by horizontal thin dotted lines were defined as mean +3 standard deviations of pre-pandemic control plasma (IgG=1160 ng/mL, IgA=283 ng/mL). For sVNT, the manufacturer's recommended cutoff of 30% is shown. DPD: Days post- qPCR positive COVID-19 diagnosis. A) IgG concentrations over time. B) IgA concentrations over time. C) Percent surrogate virus neutralization over time. D-F) Maximum titres attained per participant of IgG, IgA, and sVNT. The Kruskal-Wallis test was used to compare groups; * indicates p=0.033. Error bars show means and standard deviations. G-I) Proportions of participants who seroconverted at any point for IgG, IgA, or sVNT, defined as having a sample above the cutoff, are shown in color.

**Figure 2: Effect of HIV status and suppression on antibody neutralization capacity.** A) Neutralization of wave-concordant Beta virus and cross-neutralization of ancestral D614G and Delta virus by HIV-negative and suppressed PWH plasma. Friedman's test was used for comparing matched participant data across the different variants. * indicates p=0.033, *** p<0.001. B) Neutralization of D614G, Beta, and Delta viruses by viremic PWH plasma. C-E): Neutralization titers of HIV-negative and suppressed PWH plasma compared to viremic PWH for ancestral D614G, Beta, and Delta viruses. * indicates p=0.0499. Error bars show geometric means and geometric standard deviations. Dotted line in A-E shows the minimum tested dilution of 1:20 for the neutralization assay. Mann-Whitney test was used for comparing patient groups. F-H) Fractions of HIV-negative, suppressed PWH, and viremic PWH participants who had detectable neutralization (above limit of quantification) of ancestral D614G, Beta, and Delta viruses. The fraction of viremic PWH able to neutralize was lower with borderline significance (p=0.0499, Fisher's Exact test).

**Figure 3: Comparison of surrogate virus neutralization test (sVNT) to live virus neutralization assays (VNA).** A) First infection wave samples from participants enrolled in 2020 tested against D614G virus. B-C) Samples from participants enrolled in the Beta infection wave in early 2021, tested against

D614G (B) and Beta (C) viruses. Purple squares: HIV-negative patients; orange circles: PWH. Solid curve: sigmoidal four-parameter curve fitted to all samples; dashed curves: separate models for HIV-negative and PWH groups. Dotted lines: Positive/negative cutoff of 30% for sVNT as recommended by the manufacturer, and minimum tested dilution of 1:20 for neutralization assay.

**Figure S1: Demographic and clinical parameters affecting seroconversion.** Mantel-Haenszel rate ratios and 95% confidence intervals are shown; p-values denote statistically significant factors. A) IgG, and B) IgA.

**Figure S2: Correlations between anti-RBD antibody (IgM, IgG, and IgA) concentrations, virus neutralization assay (VNA) titers, and surrogate virus neutralization test (sVNT).** Results include samples from first and second epidemic waves. Neutralization results shown are against the D614G virus. Matrix shows Spearman correlation coefficients.

## Footnotes

### Potential conflicts of interest

All authors: No reported conflicts of interest. Chee Wah Tan and Lin-Fa Wang report the following issued patent: US patent 11054429 B1, SARS-CoV-2 surrogate virus neutralization test based on antibody-mediated blockage of ACE2-spike protein binding.

### Financial support

This work was supported by the Bill and Melinda Gates award INV-018944 (AS), National Institutes of Health award R01 AI138546 (AS), the South African Medical Research Council Award 6084COAP2020 (AS), and by a grant from the Wellcome Trust to the Africa Health Research Institute (Wellcome Trust Strategic Core Award [grant #201433/Z/16/Z]). Additional funding was received from the South Africa Research Chairs Initiative (grant #64809). Partial funding was provided by the Sub-Saharan African Network for TB/HIV Research Excellence (SANTHE), a DELTAS Africa Initiative [grant #DEL-15-006]. The DELTAS Africa Initiative is an independent funding scheme of the African Academy of Sciences (AAS)'s Alliance for Accelerating Excellence in Science in Africa (AESA) and supported by the New Partnership for Africa's Development Planning and Coordinating Agency (NEPAD Agency) with funding from the Wellcome Trust [grant #107752/Z/15/Z] and the UK government. The Duke-NUS group is supported by grants from the Singapore National Medical Research Council STPRG-FY19-001, COVID19RF-003, COVID19RF-060, MOH-000535/MOH-OFYIRG19nov-0002 and OFLCG19May-0034. The funders had no role in study design, data collection and analysis, decision to publish, or preparation of the manuscript.

## Current affiliations

S.H. Hwa's current affiliation is: Antibody Biology Unit, Laboratory of Immunogenetics, National Institute of Allergy and Infectious Diseases, Rockville, Maryland, United States. J. Snyman's current affiliation is: Department of Biochemistry, Microbiology and Immunology, College of Medicine, University of Saskatchewan, Canada.

## COMMIT-KZN Team

Adrie Steyn, University of Alabama at Birmingham, Africa Health Research Institute.

Alasdair Leslie, Africa Health Research Institute and Division of Infection and Immunity, University College London.

Dirhona Ramjit, Africa Health Research Institute.

Emily Wong, Africa Health Research Institute and Division of Infectious Diseases, University of Alabama at Birmingham.

Guy Harling, Africa Health Research Institute and the Institute for Global Health, University College London, London, UK.

Henrik Kloverpris, Africa Health Research Institute, Division of Infection and Immunity, University College London and Department of Immunology and Microbiology, University of Copenhagen.

Jackson Marakalala, Africa Health Research Institute.

Janet Seeley, Africa Health Research Institute.

Jennifer Giandhari, Kwazulu-Natal Research Innovation and Sequencing Platform.

Kaylesh Dullabh, Department of Cardiothoracic Surgery, University of KwaZulu-Natal, Durban, South Africa.

Kennedy Nyamande, Department of Pulmonology and Critical Care, University of KwaZulu-Natal, Durban, South Africa.

Kobus Herbst, Africa Health Research Institute and the South African Population Research Infrastructure Network, Durban, South Africa

Kogie Naidoo, Centre for Aids Programme of Research in South Africa, University of KwaZulu-Natal

Matilda Mazibuko, Africa Health Research Institute

Moherndran Archary, Department of Paediatrics and Child Health, University of KwaZulu-Natal, Durban, South Africa.

Mosa Moshabela, College of Health Sciences, University of KwaZulu-Natal, Durban, South Africa.

Nesri Padayatchi, Centre for Aids Programme of Research in South Africa, University of KwaZulu-Natal.

Nigel Klein, Africa Health Research Institute and the Institute of Child Health, University College London, London, UK.

Nikiwe Mbatha, Africa Health Research Institute.

Nokuthula Ngcobo, Africa Health Research Institute.

Nokwanda Gumede, Africa Health Research Institute.

Nokwanda Ngcobo, Africa Health Research Institute.

Philip Goulder, Africa Health Research Institute and Department of Paediatrics, Oxford, UK.

Prakash Jeena, Department of Paediatrics and Child Health, University of KwaZulu-Natal, Durban, South Africa.

Rajhmun Madansein, Department of Cardiothoracic Surgery, University of KwaZulu-Natal, Durban, South Africa.

Ravindra K. Gupta, Africa Health Research Institute and Cambridge Institute of Therapeutic Immunology & Infectious Disease, Cambridge, UK.

Rohen Harrichandparsad, Department of Neurosurgery, University of KwaZulu-Natal, Durban, South Africa.

Samita Singh, Africa Health Research Institute.

Thandeka Khoza, Africa Health Research Institute.

Theresa Smit, Africa Health Research Institute.

Thumbi Ndung'u, Africa Health Research Institute; Division of Infection and Immunity, University College London; HIV Pathogenesis Programme, The Doris Duke Medical Research Institute; and Max Planck Institute for Infection Biology, Berlin.

Vinod Patel, Department of Neurology, University of KwaZulu-Natal, Durban, South Africa.

Zaza Ndhlovu, Africa Health Research Institute.

## Corresponding author

Alex Sigal: alex.sigal@ahri.org. Africa Health Research Institute, 719 Umbilo Road, Congella, Durban, 4001, South Africa. Telephone: +27 31 2604589.

| | All<br>34 | HIV-<br>16 (47%) | PWH Supp.<br>7 (21%) | PWH Viremic<br>11 (32%) |
|---|---|---|---|---|
| **Age (years)*** | 41 (34 – 51) | 43 (35 – 56) | 42 (39 – 56) | 38 (28 – 42) |
| **Female** | 16 (47%) | 5 (31%) | 5 (71%) | 6 (55%) |
| **Diagnosis to first sample (days)** | 6·0 (3·8 – 8·0) | 6·0 (2·5 – 8·8) | 8·0 (4·0 – 9·0) | 5·0 (3·0 – 7·0) |
| **Moderate/severe disease**** | 10 (29%) | 3 (19%) | 3 (43%) | 4 (36%) |
| **Hypertension** | 8 (24%) | 5 (31%) | 2 (29%) | 1 (9%) |
| **Diabetes** | 4 (12%) | 3 (19%) | 1 (14%) | 0 |
| **Active TB** | 2 (6%) | 0 | 0 | 2 (18%) |
| **History of TB** | 7 (21%) | 0 | 3 (43%) | 4 (36%) |
| **ART detected***** | | | 7 (100%) | 5 (45%) |
| **Viral load (copies/mL)*** | | | <40 | 13876 (174 – 125735) |
| **CD4 (cells/µL)*** | 592 (152 – 855) | 827 (587 – 1119) | 713 (191 – 746) | 161 (9 – 453) |

**Table 1: Participant details**

ART, viral load and CD4 detected at enrollment timepoint. Supp.: Suppressed HIV viral load, <40 HIV RNA copies/mL. *Median (IQR). **Defined as requiring at least supplemental oxygen during hospitalization. ***Antiretrovirals tested: tenofovir, emtricitabine, efavirenz, dolutegravir, nevirapine, azidothymidine, abacavir, lamivudine, lopinavir, ritonavir, and atazanavir.

**Figure 1**
**281x151 mm (.26 x  DPI)**

**Figure 2**
**206x215 mm (.26 x  DPI)**

**Figure 3**

**237x175 mm (.26 x DPI)**

## Supplementary Material

jiac343_Supplementary_Data

