# Association Between Human Immunodeficiency Virus Viremia and Compromised Neutralization of Severe Acute Respiratory Syndrome Coronavirus 2 Beta Variant

**DOI:** 10.1093/infdis/jiac343

**Published:** 2022-08-17

**Authors:** Shi-Hsia Hwa, Jumari Snyman, Mallory Bernstein, Yashica Ganga, Sandile Cele, Daniel Muema, Chee Wah Tan, Khadija Khan, Farina Karim, Willem Hanekom, Leslie Bernstein, Stefan H E Kaufmann, Lin-Fa Wang, Thumbi Ndung’u, Alex Sigal, the COMMIT-KZN Team, Adrie Steyn, Alasdair Leslie, Dirhona Ramjit, Emily Wong, Guy Harling, Henrik Kloverpris, Jackson Marakalala, Janet Seeley, Jennifer Giandhari, Kaylesh Dullabh, Kennedy Nyamande, Kobus Herbst, Kogie Naidoo, Matilda Mazibuko, Moherndran Archary, Mosa Moshabela, Nesri Padayatchi, Nigel Klein, Nikiwe Mbatha, Nokuthula Ngcobo, Nokwanda Gumede, Nokwanda Ngcobo, Philip Goulder, Prakash Jeena, Rajhmun Madansein, Ravindra K Gupta, Rohen Harrichandparsad, Samita Singh, Thandeka Khoza, Theresa Smit, Max Planck, Vinod Patel, Zaza Ndhlovu

**Affiliations:** Africa Health Research Institute, Durban, South Africa; Division of Infection and Immunity, University College London, London, United Kingdom; Africa Health Research Institute, Durban, South Africa; HIV Pathogenesis Programme, University of KwaZulu-Natal, Durban, South Africa; School of Laboratory Medicine and Medical Sciences, University of KwaZulu-Natal, Durban, South Africa; Africa Health Research Institute, Durban, South Africa; Africa Health Research Institute, Durban, South Africa; Africa Health Research Institute, Durban, South Africa; School of Laboratory Medicine and Medical Sciences, University of KwaZulu-Natal, Durban, South Africa; Africa Health Research Institute, Durban, South Africa; HIV Pathogenesis Programme, University of KwaZulu-Natal, Durban, South Africa; School of Laboratory Medicine and Medical Sciences, University of KwaZulu-Natal, Durban, South Africa; Programme in Emerging Infectious Diseases, Duke-NUS Medical School, Singapore, Singapore; Africa Health Research Institute, Durban, South Africa; School of Laboratory Medicine and Medical Sciences, University of KwaZulu-Natal, Durban, South Africa; Africa Health Research Institute, Durban, South Africa; School of Laboratory Medicine and Medical Sciences, University of KwaZulu-Natal, Durban, South Africa; Africa Health Research Institute, Durban, South Africa; Division of Infection and Immunity, University College London, London, United Kingdom; Department of Population Sciences, Beckman Research Institute, City of Hope Comprehensive Cancer Center, Duarte, California, USA; Max Planck Institute for Infection Biology, Berlin, Germany; Max Planck Institute for Multidisciplinary Sciences, Göttingen, Germany; Hagler Institute for Advanced Study, Texas A&M University, College Station, Texas, USA; Programme in Emerging Infectious Diseases, Duke-NUS Medical School, Singapore, Singapore; SingHealth Duke-NUS Global Health Institute, Singapore, Singapore; Africa Health Research Institute, Durban, South Africa; Division of Infection and Immunity, University College London, London, United Kingdom; HIV Pathogenesis Programme, University of KwaZulu-Natal, Durban, South Africa; School of Laboratory Medicine and Medical Sciences, University of KwaZulu-Natal, Durban, South Africa; Ragon Institute of Massachusetts General Hospital, Massachusetts Institute of Technology and Harvard University, Cambridge, Massachusetts, USA; Africa Health Research Institute, Durban, South Africa; School of Laboratory Medicine and Medical Sciences, University of KwaZulu-Natal, Durban, South Africa; Max Planck Institute for Infection Biology, Berlin, Germany; Centre for the AIDS Programme of Research in South Africa, Durban, South Africa

**Keywords:** SARS-CoV-2, COVID-19, HIV, antibodies, antiretroviral therapy, Beta variant, neutralization

## Abstract

**Background:**

Severe acute respiratory syndrome coronavirus 2 (SARS-CoV-2) infection may be associated with worse clinical outcomes in people with human immunodeficiency virus (HIV) (PWH). We report anti–SARS-CoV-2 antibody responses in patients hospitalized with coronavirus disease 2019 in Durban, South Africa, during the second SARS-CoV-2 infection wave dominated by the Beta (B.1.351) variant.

**Methods:**

Thirty-four participants with confirmed SARS-CoV-2 infection were followed up with weekly blood sampling to examine antibody levels and neutralization potency against SARS-CoV-2 variants. Participants included 18 PWH, of whom 11 were HIV viremic.

**Results:**

SARS-CoV-2–specific antibody concentrations were generally lower in viremic PWH than in virologically suppressed PWH and HIV-negative participants, and neutralization of the Beta variant was 4.9-fold lower in viremic PWH. Most HIV-negative participants and antiretroviral therapy–suppressed PWH also neutralized the Delta (B.1.617.2) variant, whereas the majority of viremic PWH did not. CD4 cell counts <500/μL were associated with lower frequencies of immunoglobulin G and A seroconversion. In addition, there was a high correlation between a surrogate virus neutralization test and live virus neutralization against ancestral SARS-CoV-2 virus in both PWH and HIV-negative individuals, but correlation decreased for the Beta variant neutralization in PWH.

**Conclusions:**

HIV viremia was associated with reduced Beta variant neutralization. This highlights the importance of HIV suppression in maintaining an effective SARS-CoV-2 neutralization response.

## Teaser

People with human immunodeficiency virus viremia elicited lower neutralizing antibody titers against infection with the Beta variant of severe acute respiratory syndrome coronavirus 2 and the majority were unable to cross-neutralize the Delta variant.

The second epidemic wave of coronavirus disease 2019 (COVID-19) in South Africa was dominated by the Beta variant of concern (20H/501Y.V2, Pango lineage B.1.351) which emerged in the Eastern Cape Province. By mid-November 2020, Beta represented the majority of sequenced samples [1]. Spike mutations in the receptor-binding domain (RBD) and N-terminal domain result in partial antigenic escape of Beta from neutralizing antibody immunity elicited by ancestral strains [2, 3], and the efficacy of the ChAdOx1 nCoV-19 vaccine in preventing mild to moderate COVID-19 dropped from 75% before 31 October 2021 to 10% when the Beta variant became prevalent [4].

People with human immunodeficiency virus (HIV) (PWH) may be at higher risk for death from COVID-19 [5] and for more severe COVID-19 outcomes [6–8]. This may be owing to an impaired T-cell and antibody response to severe acute respiratory syndrome coronavirus 2 (SARS-CoV-2) infection in PWH [9], as neutralizing antibodies are correlated with vaccine efficacy and protection against COVID-19 [10]. We found no differences in the antibody responses of COVID-19 PWH versus HIV-negative participants in the first infection wave in South Africa before emergence of variants of concern [11]. However, our group observed higher disease severity in PWH in our cohort of infected, unvaccinated participants during the Beta (but not the ancestral virus) infection wave [6]. Therefore, we reexamined antibody neutralizing immunity in PWH in the Beta infection wave.

In the current study, we evaluated whether, during the second infection wave dominated by the Beta variant, PWH differed in their infection-elicited antibody responses to SARS-CoV-2. We measured isotype-specific spike RBD-binding and virus neutralizing antibody responses within the first 60 days after COVID-19 diagnosis in PWH and HIV-negative participants. We also evaluated the suitability of a commercial surrogate virus neutralization test (sVNT) in this patient population [12]. In agreement with our group’s previous reports showing more severe COVID-19 infection outcomes and altering of immune responses in PWH in the Beta-dominated second infection wave in South Africa [6] and lower levels of Delta (B.1.617.2) neutralization capacity in unvaccinated PWH [13], we observed lower Beta infection–elicited neutralization capacity of the Beta variant in PWH with detectable HIV viremia.

## METHODS

### Ethical Statement and Study Participants

The study location and sampling methodology have been described elsewhere [6]. The study protocol was approved by the University of KwaZulu-Natal Biomedical Research Ethics Committee (reference BREC/00001275/2020). Written informed consent was obtained for all enrolled participants. Hospitalized patients with SARS-CoV-2 infection in Durban, KwaZulu-Natal, South Africa, were enrolled in the study and followed up weekly with collection of oropharyngeal/nasopharyngeal swab and whole-blood samples at each study visit. Inclusion criteria were SARS-CoV-2 infection confirmed by reverse transcription-quantitative polymerase chain reaction (RT-qPCR) and age >18 years. All participants meeting inclusion criteria were eligible for enrollment.

For analyses of antibody responses, we selected participants who had been enrolled during the second, Beta-dominated COVID-19 infection wave in South Africa and had a baseline blood sample at enrollment and ≥1 additional sample covering the first month after the date of the diagnostic swab sample (dates of diagnosis for COVID-19 ranged from 30 December 2020 to 1 April 2021). None of the participants were vaccinated at the time of sample collection. Because the date of symptom onset depended on recall, which may vary across participants, we used days after diagnostic swab sample for longitudinal analyses. Eighteen PWH were available within that time period, and we arbitrarily selected the first 16 HIV-negative participants who also fit these criteria in order to have a similar number of controls. COVID-19 vaccines had not yet been made available to the general population in South Africa during the study period, and none of the participants had been vaccinated during the sampling period included in these analyses.

### Laboratory Testing

RT-qPCR for SARS-CoV-2 ORF1ab, S, and N genes was performed. Commercial diagnostic laboratories in Durban, South Africa, performed testing for HIV viral load (Molecular Diagnostic Services, and CD4 and CD8 cell counts (Ampath). We defined viremia as any viral load above the limit of detection of 40 copies/mL. The presence of antiretroviral therapy (ART) components in plasma of PWH was measured using liquid chromatography with tandem mass spectrometry [6].

### Enzyme-Linked Immunosorbent Assay

Isotype-specific RBD enzyme-linked immunosorbent assays were performed as described elsewhere [11]. Briefly, plates were coated with recombinant SARS-CoV-2 RBD (gift from Galit Alter), blocked, and incubated with plasma sample dilutions. Secondary (detection) antibodies for immunoglobulin (Ig) G, IgM, and IgA were isotype-specific, cross-adsorbed, horseradish-peroxidase–conjugated polyclonal antibodies. For each isotype, an RBD-binding monoclonal antibody was used to generate a standard curve for interpolating concentrations of anti-RBD–specific antibodies, namely, CR3022 IgG (gift from Galit Alter), hIgM2001 (GenScript), and hIgA2001 (GenScript). Prepandemic plasma from HIV-uninfected individuals and commercial human serum (European Union/US origin; BioWest) were used to establish baselines per isotype, as described elsewhere [11].

### sVNT Protocol

An sVNT based on detecting inhibition of recombinant human angiotensin-converting enzyme 2 (ACE2) binding to RBD-peroxidase fusion protein was performed according to the manufacturer’s instructions (GenScript SARS-CoV-2 sVNT; version RUO 3.0). All samples were tested at a single dilution of 1:10. Sample results are reported as the percentage of inhibition relative to the kit negative control, with a manufacturer-recommended positive cutoff value of ≥30%.

### Cells

Vero E6 cells (American Type Culture Collection CRL-1586) were obtained from Cellonex in South Africa and propagated as described elsewhere [3]. An in-house cell line, H1299-ACE2, was generated by infecting H1299 cells (American Type Culture Collection CRL-5803) with an ACE2-overexpressing stable lentiviral vector [3].

### Viruses

SARS-CoV-2 D614G, Beta, and Delta isolates used in these experiments are described in our group’s previous work [3, 14]. Passage 3 stocks were used. All work with live virus was performed in biosafety level 3 containment, using protocols for SARS-CoV-2 approved by the Africa Health Research Institute Biosafety Committee.

### Virus Neutralization Assay

Authentic virus neutralization assays of plasma antibodies based on reduction of immunostained focus-forming units per well were performed using a similar procedure as in our group’s previous work [3, 13–15], with the following modifications owing to the larger size of Beta virus foci: we reduced the input viral load to 70 focus-forming units per well and shortened the incubation time to 18 hours after infection for all 3 D614G (first wave), Beta, and Delta isolates to minimize overlapping foci. Plates were fixed, stained, scanned, and counted as described elsewhere [3].

Focus counts per well were normalized against the average of the no-antibody virus control wells on each plate. The 50% focus reduction neutralization titer (FRNT_50_) expressed as the inverse of the sample dilution was calculated using Prism software by fitting normalized focus counts for each sample to the 4-parameter Hill equation, with the bottom and top parameters constrained to a range of 0–1. These included extrapolated values for a few samples that had marginally detectable neutralization at the lowest tested dilution of 1:20. Samples with no neutralization at all were assigned a value of 1 (0 log_10_). A rabbit monoclonal antibody BS-R2B2 (GenScript A02051) was included as a positive control in each run. The FRNT_50_ of BS-R2B2 was 7.4 ng/mL against D614G and 5.0 ng/mL against Beta virus.

### Statistical Analysis

Prism (version 9; GraphPad) and Stata (version 17; StataCorp) software were used for data analysis. Standard statistical methods, including χ^2^, Fisher exact, Friedman, Kruskal-Wallis, and Mann-Whitney *U* tests were used to compare groups and estimate relationships between variables. To compare the sVNT versus neutralization assay, a 4-parameter logistic model with bottom and top constrained to 0%–100% was used because the sVNT result is given as the percentage of inhibition relative to assay controls. To examine the effects of clinical factors on antibody seroconversion and loss, we used Mantel-Haenszel methods to determine univariate and multivariate-adjusted rate ratios (RRs) and corresponding 95% confidence intervals (CIs). Results presented here are univariate because sample size was a limitation for multivariate adjustment and multivariate results. Differences were considered statistically significant at *P* < .05, and all statistical tests are 2 sided.

## RESULTS

Participants in the current study had RT-qPCR–confirmed SARS-CoV-2 infection during the Beta infection wave in South Africa [14], with dates of diagnosis ranging from the end of December 2020 to the start of April 2021. No study participants were vaccinated at the time of collection, and to the best of our knowledge immunity measured here resulted from SARS-CoV-2 infection in the second infection wave in South Africa. Samples from 34 participants were analyzed, and these participants included 18 PWH (53%) and 16 HIV-negative participants (Table 1). Seven of the 18 PWH had a history of tuberculosis (*P* = .008; Fisher exact test). Eleven of the 18 PWH were viremic, and 13 had CD4 cell counts >500/μL on enrollment. Median CD4 counts were significantly lower in HIV-viremic than in HIV-suppressed PWH (viremic PWH, 161/μL [interquartile range, 9–453/μL]; suppressed PWH, 713/μL [191–746/μL]; *P* = .04). Five of 11 (45%) viremic PWH and all HIV-suppressed PWH had detectable ART at enrollment. Disease severity was higher in the PLW group, but this difference was not statistically significant.

**Table 1.** Characteristics of Study Participants

Characteristic	Participants, No. (%)^a^			
	All Participants (n = 34)	HIV-Negative Participants (n = 16)	HIV-Suppressed PWH (n = 7)^b^	HIV-Viremic PWH (n = 11)
Age, median (IQR), y	41 (34–51)	43 (35–56)	42 (39–56)	38 (28–42)
Female sex	16 (47)	5 (31)	5 (71)	6 (55)
Time from diagnosis to 1st sample, median (IQR), d	6.0 (3.8–8.0)	6.0 (2.5–8.8)	8.0 (4.0–9.0)	5.0 (3.0–7.0)
Moderate or severe disease^c^	10 (29)	3 (19)	3 (43)	4 (36)
Hypertension	8 (24)	5 (31)	2 (29)	1 (9)
Diabetes	4 (12)	3 (19)	1 (14)	0
Active tuberculosis	2 (6)	0	0	2 (18)
History of tuberculosis	7 (21)	0	3 (43)	4 (36)
ART detected^d,e^	…	…	7 (100)	5 (45)
Viral load, median (IQR), copies/mL^d^	…	…	<40	13876 (174–125735)
CD4 cell count, median (IQR), cells/μL^d^	592 (152–855)	827 (587–1119)	713 (191–746)	161 (9–453)

Abbreviations: ART, antiretroviral therapy; HIV, human immunodeficiency virus; IQR, interquartile range; PWH, people with HIV.

Data represent no. (%) of participants unless otherwise specified.

HIV suppression defined as viral load <40 HIV RNA copies/mL.

Moderate or severe disease was defined as requiring at least supplemental oxygen during hospitalization.

ART, viral load, and CD4 cell count determined at enrollment.

Antiretrovirals tested for included tenofovir, emtricitabine, efavirenz, dolutegravir, nevirapine, azidothymidine, abacavir, lamivudine, lopinavir, ritonavir, and atazanavir.

**Figure 1.**
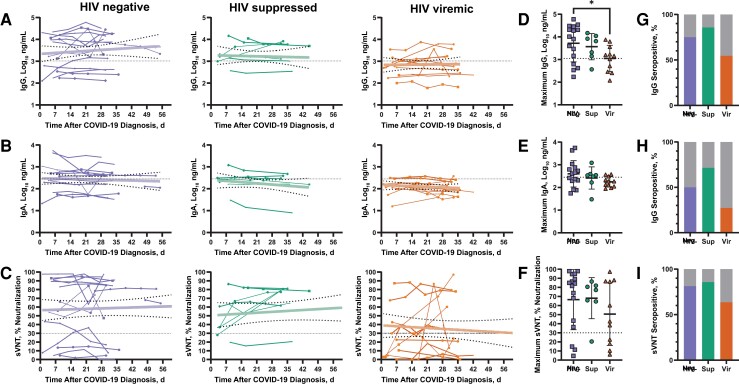
Effect of human immunodeficiency virus (HIV) status and suppression on severe acute respiratory syndrome coronavirus 2 anti-spike receptor-binding domain (RBD) antibodies. Anti-RBD antibody concentrations and surrogate virus neutralization test (sVNT) values in study participants with coronavirus disease 2019 (COVID-19), including HIV-negative individuals (*first column*) and virologically suppressed (*second column*) and viremic (*third column*) people with HIV. Individual participants’ data points are shown. Linear trends in pooled data are shown as transparent ribbons; 95% confidence intervals, as thick dotted lines. For immunoglobulin (Ig) G and IgA, baseline cutoffs, indicated by horizontal thin dotted lines, were defined as means plus 3 standard deviations of prepandemic control plasma concentrations (IgG, 1160 ng/mL; IgA, 283 ng/mL). For sVNT, the manufacturer’s recommended cutoff of 30% is shown. COVID-19 was diagnosed by means of reverse-transcription quantitative polymerase chain reaction. *A–C,* IgG and IgA concentrations and percentage of surrogate virus neutralization over time. *D–F,* Maximum IgG and IgA concentrations and sVNT titers per participant. Error bars show means and standard deviations. *P = .03 (Kruskal-Wallis test). Abbreviations: Neg, HIV negative; Sup, HIV suppressed; Vir, HIV viremic. *G–I,* Proportions of participants who seroconverted at any point for IgG, IgA, or sVNT, defined as having a sample above the cutoff, shown in color.

We monitored changes in SARS-CoV-2 spike RBD antibody levels weekly up until about 1 month after enrollment, where enrollment was visit 1, and there were 5 visits over the 1-month period. An additional collection was performed after the 1-month period if the participant was available (visit 6). Collection points per participant are graphed in Figure 1. The majority of the 34 participants in this analysis provided samples at weekly follow-up visits 2, 3, 4, and 5 (25, 25, 27, and 31 participants respectively), and 13 participants provided samples at visit 6. For IgG (Figure 1*A*), IgA (Figure 1*B*), and sVNT (Figure 1*C*), responses, results from viremic PWH trended lower compared with HIV-negative individuals and HIV-suppressed PWH. IgG and sVNT responses were already above assay cutoff values (IgG, 1160 ng/mL; sVNT, 30%) at the earliest time point sampled for the majority of HIV-negative individuals (69% for IgG and 75% for sVNT) and HIV-suppressed PWH (71% and 71%, respectively) but not for viremic PWH (18% and 36%). The maximum IgG concentration attained during the sampling period was higher in HIV-negative individuals than in viremic PWH (*P* = .03), but no other significant differences in maximum antibody titers were observed (Figure 1*D*–1*F*). Proportions of HIV-viremic individuals who seroconverted at any point in these 3 assays were lower than the proportions of HIV-suppressed or HIV-negative individuals (Figure 1*G*–1*I*); however, these differences were not significant.

**Figure 2.**
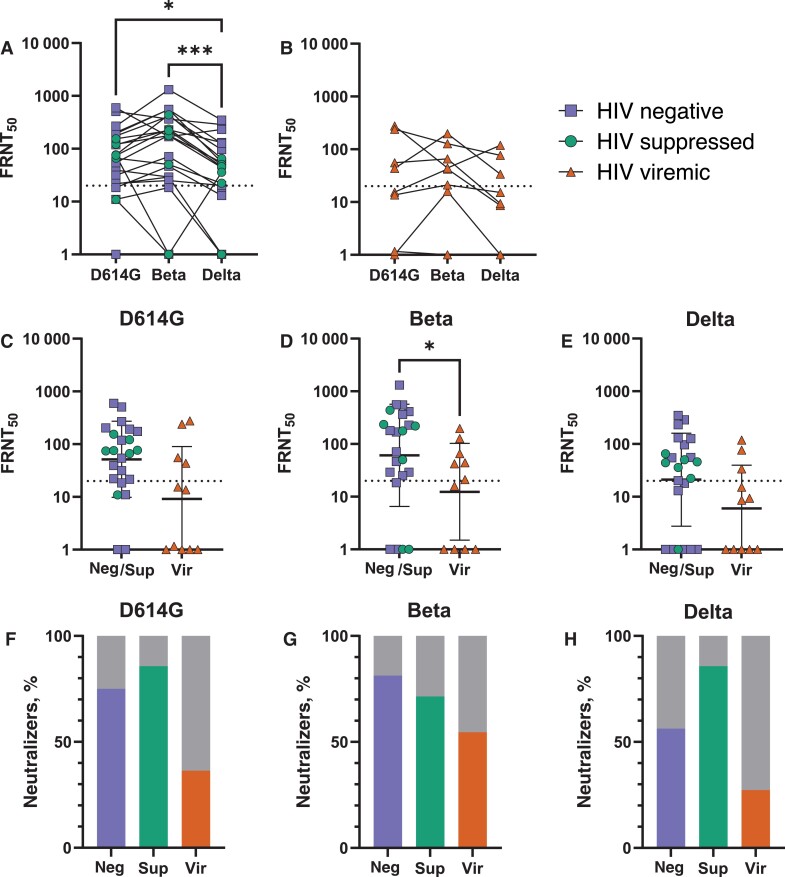
Effect of human immunodeficiency virus (HIV) status and suppression on antibody neutralization capacity. *A*, Neutralization of wave-concordant Beta virus and cross-neutralization of ancestral D614G and Delta virus by plasma samples from HIV-negative individuals and HIV-suppressed people with HIV (PWH). Friedman’s test was used to compare matched participant data across the different variants. *P = .03; ***P < .001. Abbreviation: FRNT_50_, 50% focus reduction neutralization titer. *B,* Neutralization of D614G, Beta, and Delta viruses by plasma samples from viremic PWH. *C*–*E,* Neutralization titers of plasma samples from HIV-negative participants (Neg) and HIV-suppressed PWH (Sup) compared with viremic PWH (Vir) for ancestral D614G, Beta, and Delta viruses. Error bars show geometric means and geometric standard deviations. Dotted lines in *A­–E* show the minimum tested dilution of 1:20 for the neutralization assay. *P = .0499; Mann-Whitney test was used to compare patient groups. *F*–*H,* Fractions of HIV-negative individuals and HIV-suppressed and viremic PWH who had detectable neutralization (above limit of quantification) of ancestral D614G, Beta, and Delta viruses. The fraction of viremic PWH able to neutralize was lower but with borderline significance (*P* = .0499; Fisher exact test).

Virus neutralization assays were conducted for the closest available sample to 1 month after diagnosis per participant (median, 29 days [interquartile range, 24–33 days]) against live virus isolates of D614G (first infection wave/ancestral), Beta (same infection wave), and Delta (following wave). Given that there were no detectable differences between HIV-negative and HIV-suppressed participants (Figure 1), neutralization of different variants/strains was compared in a combined group of HIV-negative individuals and HIV-suppressed PWH (Figure 2*A*) and in HIV-viremic PWH (Figure 2*B*) to increase statistical power. The geometric mean titer FRNT_50_ in HIV-negative and HIV-suppressed participants was 51.7 against D614G virus, 60.9 against Beta virus, and 21.1 against Delta virus, slightly above the assay limit of quantification of 1:20 minimum tested dilution (Figure 2*A*). The FRNT_50_ geometric mean titers against all 3 variants of plasma from viremic PWH were below the limit of quantification (Figure 2*B*).

Relative to HIV-negative and HIV-suppressed participants, those who were HIV viremic showed a trend toward lower neutralization of the ancestral strain (Figure 2*C*), the Beta variant (Figure 2*D*), and the Delta variant (Figure 2*E*); this was significant for the Beta variant. However, the exact fold change was difficult to determine because neutralization capacity in multiple viremic participants was below the limit of quantification (LOQ) of a 1:20 plasma dilution. Proportions of HIV-viremic PWH who had quantifiable neutralization, defined as titers above LOQ at the time point 1 month after diagnosis, trended lower relative to HIV-suppressed PWH and HIV-negative participants for all 3 variants (Figure 2*F*–2*H*); this difference had borderline significance for Delta (had neutralization above LOQ, 56% of HIV-negative participants vs 86% of suppressed and 27% of viremic PWH; *P* = .049 [χ^2^ test]).

We investigated associations between participant parameters and antibody levels (Supplementary Figure 1). Moderate/severe COVID-19, defined as at least requiring supplemental oxygen, was not significantly associated with antibody levels. Age ≥45 years was significantly associated with higher concentrations of IgG and IgA, as well as higher RRs based on the Mantel-Haenszel method (RR, 3.8 for IgG and 4.1 for IgA). Male participants had a lower rate of IgG seroconversion than females (RR, 0.40). CD4 cell counts <500/μL were associated with lower frequencies of IgG (Fisher exact test *P* = .04) and IgA (*P* = .02) seroconversion. Lower rates of IgA seroconversion were associated with both HIV viremia (RR, 0.35) and CD4 counts <500 (0.34). Participants with a previous tuberculosis diagnosis had higher rate of IgA seroconversion (RR, 3.2).

**Figure 3.**
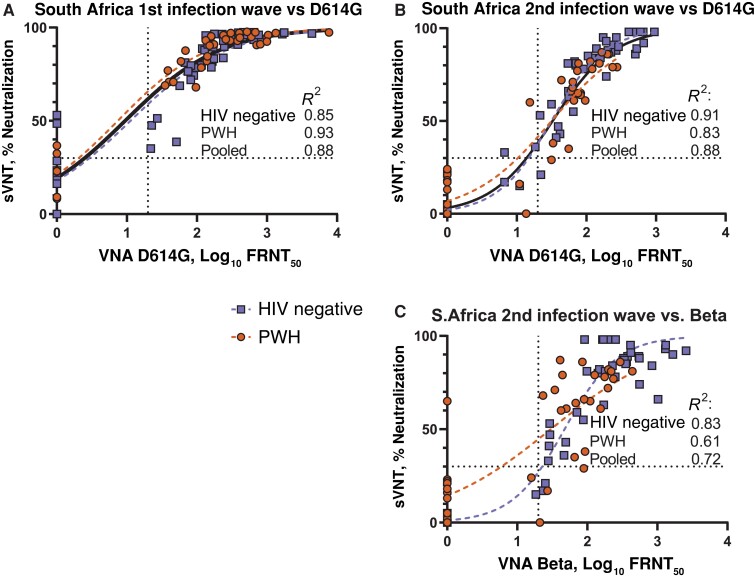
Comparison of surrogate virus neutralization test (sVNT) with live virus neutralization assays (VNAs). *A,* First infection wave samples from participants enrolled in 2020 tested against D614G virus. *B*, *C,* Samples from participants enrolled in the Beta infection wave in early 2021, tested against D614G (*B*) and Beta (*C*) viruses. Squares represent human immunodeficiency virus (HIV)–negative individuals; circles, people with HIV (PWH); solid curve, sigmoidal 4-parameter curve fitted to all samples; dashed curves, separate models for HIV-negative and PWH groups; dotted lines, positive/negative cutoff of 30% for sVNT as recommended by the manufacturer and minimum tested dilution of 1:20 for neutralization assay. Abbreviation: FRNT_50_, 50% focus reduction neutralization titer.

We also compared the sVNT with the authentic virus neutralization assay, including the first-wave plasma samples described elsewhere [11]. Compared with virus neutralization of D614G virus, coefficients of determination were similar for first-and second-wave participants (*R*^2^, 0.88 and 0.88, respectively) (Figure 3*A* and 3*B*). Model fit differed significantly between HIV-negative participants and PWH only for Beta virus neutralization by Beta infection wave samples (*P* = .01) (Figure 3*C*). Goodness of fit was lower when comparing the sVNT with Beta virus neutralization (*R*^2^ = 0.61 for PWH and 0.83 for HIV-negative patients) (Figure 3*C*). The false-positive rate for sVNT compared with virus neutralization was 5.1% (4 of 79) for samples from the first (ancestral) infection wave versus D614G, 3.2% (3 of 95) for samples from the second (Beta) infection wave versus D614G, and 1.1% (1 of 95) for second-wave samples versus Beta.

Finally, we compared the enzyme-linked immunosorbent assay, virus neutralization (for D614G), and sVNT results across all first- and second-wave samples (Supplementary Figure 2). IgG concentration was most strongly correlated with virus neutralization titers and percentage of surrogate neutralization inhibition.

## DISCUSSION

We found that HIV viremia attenuates antibody neutralization capacity elicited by Beta variant infection. These results contrast with our findings on participants infected with ancestral SARS-CoV-2 during the first infection wave in South Africa, where no statistically significant differences in antibody responses were found between PWH and HIV-negative participants [11]. However, a much smaller proportion of patients in the first-wave study were viremic. We note that the proportion of viremic participants doubled in the second infection wave [6]. The higher number of HIV viremic participants may explain the differing effect of HIV on antibody responses between the first and second infection waves, although Beta variant-specific factors should not be ruled out.

At 1 month after diagnosis, the viremic PWH group, more than half of whom showed no detectable antiretrovirals in the blood, showed a lower frequency of sampled timepoints where detectable SARS-CoV-2 neutralization was present as well as lower mean neutralization titers. When the samples were collected, vaccines were not yet available to the general population in South Africa (see https://sacoronavirus.co.za/latest-vaccine-statistics/ for vaccine administration over time in South Africa) and the study participants were unvaccinated. Furthermore, reinfection by the Beta variant in the second infection wave among people previously infected with ancestral virus in the first South African wave was reportedly rare [16]. Therefore, the effect of HIV viremia on Beta neuralization capacity is measured here in a relatively homogeneous group of participants with likely no previous SARS-CoV-2 immunity.

Against the Delta variant, plasma samples from HIV-negative participants showed a statistically significant decrease in neutralization relative to earlier variants, similar to previous findings by us and others [14, 17, 18]. The majority of viremic PWH could not neutralize Delta and therefore may have even lower protection. Impaired CD4- and IgG-specific responses to SARS-CoV-2 antigens have also been observed in PWH with active tuberculosis [9]. Other arms of the immune system, such as CD8 T cells, may offer cross-protection from newer variants such as Omicron, as most CD8 epitopes in the S protein appear to be conserved in HIV-negative donors [19, 20]; however, this remains to be determined in PWH.

In our group’s previous work, we showed that impaired neutralization of the Delta variant in COVID-19 convalescent PWH mostly affected persons with suboptimal HIV suppression [13], consistent with another study [21]. Among PWH with low/undetectable HIV viral loads who were vaccinated with ChAdOx1 nCoV-19 adenoviral vectored vaccine or BNT162b2 messenger RNA vaccine, robust anti-S and neutralizing antibody responses developed [4, 22–24]. In contrast, case reports of PWH with advanced HIV disease and low CD4 T-cell counts showed reduced antibody responses, delayed SARS-CoV-2 clearance, SARS-CoV-2 evolution of escape mutations, and a poor response to vaccination [14, 25–27]. In a cohort study in which 11.7% of PWH were viremic, PWH overall had lower anti-RBD IgG concentrations and sVNT titers than HIV-negative subjects, although the authors did not stratify by viremia [7].

During the pandemic, several countries, including South Africa, have reported decreases in HIV testing, ART initiation, or adherence to ART for various reasons, including stress on healthcare systems, lockdowns, and global disruptions to shipping and drug supplies [28]. Our group documented lower ART coverage and an increase in the frequency of HIV viremia among patients hospitalized with COVID-19 and enrolled in our cohort during the Beta variant infection wave [6]. SARS-CoV-2 spike mutations resembling variants of concern have also been observed to evolved in immunosuppression owing to advanced HIV and other immunosuppressed conditions [14, 29].

Finally, we have shown that a surrogate neutralization test that measures blocking of the S RBD­–human ACE2 interaction correlated well with the live virus neutralization assay in South African convalescent plasma samples, including from PWH, although the correlation was lower in samples obtained during the Beta infection wave, and this difference was most pronounced for PWH. The reason for this is unclear. Reasons may include a shift in the binding of neutralizing antibodies away from the RBD in Beta variant–infected PWH, which makes the RBD region tested by sVNT less representative of the neutralization response overall. We have previously observed that Beta variant infection leads to an antibody response that is more concentrated on residues 443–452 of the spike RBD and less affected by mutations at residue 484 relative to ancestral virus–elicited immunity [30]. It is possible that such shifts are not limited to the RBD and may include shifts to other domains, such as the spike N-terminal domain [31]. The focus on the RBD may be a limitation of the sVNT approach.

A limitation to the study is the small number of samples, because of the logistics of sample collection during lockdown due to the Beta epidemic wave in South Africa. Of the total 92 participants enrolled during the second-wave study period, only the 34 included in the current analysis were available for blood sampling 1 month after the diagnostic swab sample. This may have been a result of the strict lockdown, which limited mobility after discharge.

The small sample size in this study may have made the higher COVID-19 disease severity our group observed previously between PWH and HIV-negative participants [6] statistically nonsignificant. Increased disease severity is correlated with higher antibody levels and neutralization capacity [32], yet we measured lower neutralization capacity in viremic PWH. If disease severity is indeed higher in this group of PWH, it may indicate that we are underestimating the interference of HIV viremia with development of neutralization capacity to Beta variant infection. We may also be underestimating the attenuation of neutralization capacity due to suppressed HIV infection, since we detected little difference between HIV-suppressed and HIV-negative participants despite possible increased disease severity in the HIV-suppressed group.

To conclude, we have found that HIV infection that is not effectively suppressed by ART compromises the neutralizing antibody response to SARS-CoV-2 in the South African population. This shows that the level of HIV suppression, not HIV status alone, may modulate the neutralizing immune response to SARS-CoV-2 variants. ART administration and adherence is key to protecting PWH from adverse outcomes with SARS-CoV-2 infection.

## Supplementary Data

Supplementary materials are available at *The Journal of Infectious Diseases* online. Consisting of data provided by the authors to benefit the reader, the posted materials are not copyedited and are the sole responsibility of the authors, so questions or comments should be addressed to the corresponding author.

## Notes

***Acknowledgments.*** We thank Galit Alter (Ragon Institute) for the receptor-binding domain and CR3022 antibody used in the enzyme-linked immunosorbent assays, as well as the draft enzyme-linked immunosorbent assays protocol, which we used as a basis for developing our version. We also thank Katya Govender and John Adamson at the Africa Health Research Institute for liquid chromatography with tandem mass spectrometry analysis.

***COMMIT-KZN Team.*** Team members include the following: Adrie Steyn (University of Alabama at Birmingham, and Africa Health Research Institute [AHRI], Durban, South Africa), Alasdair Leslie (AHRI and Division of Infection and Immunity, University College London, London, UK), Dirhona Ramjit (AHRI), Emily Wong (AHRI and Division of Infectious Diseases, University of Alabama at Birmingham, Birmingham, Alabama), Guy Harling (AHRI and the Institute for Global Health, University College London). Henrik Kloverpris (AHRI, Division of Infection and Immunity, University College London, and Department of Immunology and Microbiology, University of Copenhagen, Copenhagen, Denmark), Jackson Marakalala (AHRI), Janet Seeley (AHRI), Jennifer Giandhari (KwaZulu-Natal Research Innovation and Sequencing Platform, Durban, South Africa), Kaylesh Dullabh (Department of Cardiothoracic Surgery, University of KwaZulu-Natal, Durban), Kennedy Nyamande (Department of Pulmonology and Critical Care, University of KwaZulu-Natal), Kobus Herbst (AHRI and the South African Population Research Infrastructure Network, Durban), Kogie Naidoo (Centre for Aids Programme of Research in South Africa, University of KwaZulu-Natal), Matilda Mazibuko (AHRI), Moherndran Archary (Department of Paediatrics and Child Health, University of KwaZulu-Natal), Mosa Moshabela (College of Health Sciences, University of KwaZulu-Natal), Nesri Padayatchi (Centre for Aids Programme of Research in South Africa, University of KwaZulu-Natal), Nigel Klein (AHRI and the Institute of Child Health, University College London), Nikiwe Mbatha (AHRI), Nokuthula Ngcobo (AHRI), Nokwanda Gumede (AHRI), Nokwanda Ngcobo (AHRI), Philip Goulder (AHRI and Department of Paediatrics, University of Oxford, Oxford, UK), Prakash Jeena (Department of Paediatrics and Child Health, University of KwaZulu-Natal), Rajhmun Madansein (Department of Cardiothoracic Surgery, University of KwaZulu-Natal), Ravindra K. Gupta (AHRI and Cambridge Institute of Therapeutic Immunology & Infectious Disease, Cambridge, UK), Rohen Harrichandparsad (Department of Neurosurgery, University of KwaZulu-Natal), Samita Singh (AHRI), Thandeka Khoza (AHRI), Theresa Smit (AHRI); and Max Planck Institute for Infection Biology, Berlin, Germany), Vinod Patel (Department of Neurology, University of KwaZulu-Natal), and Zaza Ndhlovu (AHRI).

***Disclaimer.*** The funders had no role in the study design, data collection and analysis, decision to publish, or preparation of the manuscript.

***Financial support.*** This work was supported by the Bill & Melinda Gates Foundation (award INV-018944 to A. S.); the National Institutes of Health (award R01 AI138546 to A. S.); the South African Medical Research Council (award 6084COAP2020 to A. S.); the Wellcome Trust (Wellcome Trust Strategic Core Award 201433/Z/16/Z to the Africa Health Research Institute); the South Africa Research Chairs Initiative (grant 64809); the Singapore National Medical Research Council (grants STPRG-FY19-001, COVID19RF-003, COVID19RF-060, MOH-000535/MOH-OFYIRG19nov-0002, and OFLCG19May-0034 to the Duke-NUS group). This study was also funded as part of the DELTAS Africa Initiative grant to the Sub-Saharan African Network for TB/HIV Research Excellence (SANTHE), where the DELTAS Africa initiative is an independent funding scheme of the African Academy of Sciences Alliance for Accelerating Excellence in Science in Africa (AESA) with funding from the Wellcome Trust [grant #107752/Z/15/Z]. Funding to pay the Open Access publication charges for this article was provided by internal Africa Health Research Institute funding to A.S.

## Supplementary Material

jiac343_Supplementary_Data
