# Supplementary figures and images for: Association Between Human Immunodeficiency Virus Viremia and Compromised Neutralization of Severe Acute Respiratory Syndrome Coronavirus 2 Beta Variant
Source: J Infect Dis. 2022 Aug 17;227(2):211–20. doi: 10.1093/infdis/jiac343 (PMC9452105; doi:10.1093/infdis/jiac343)

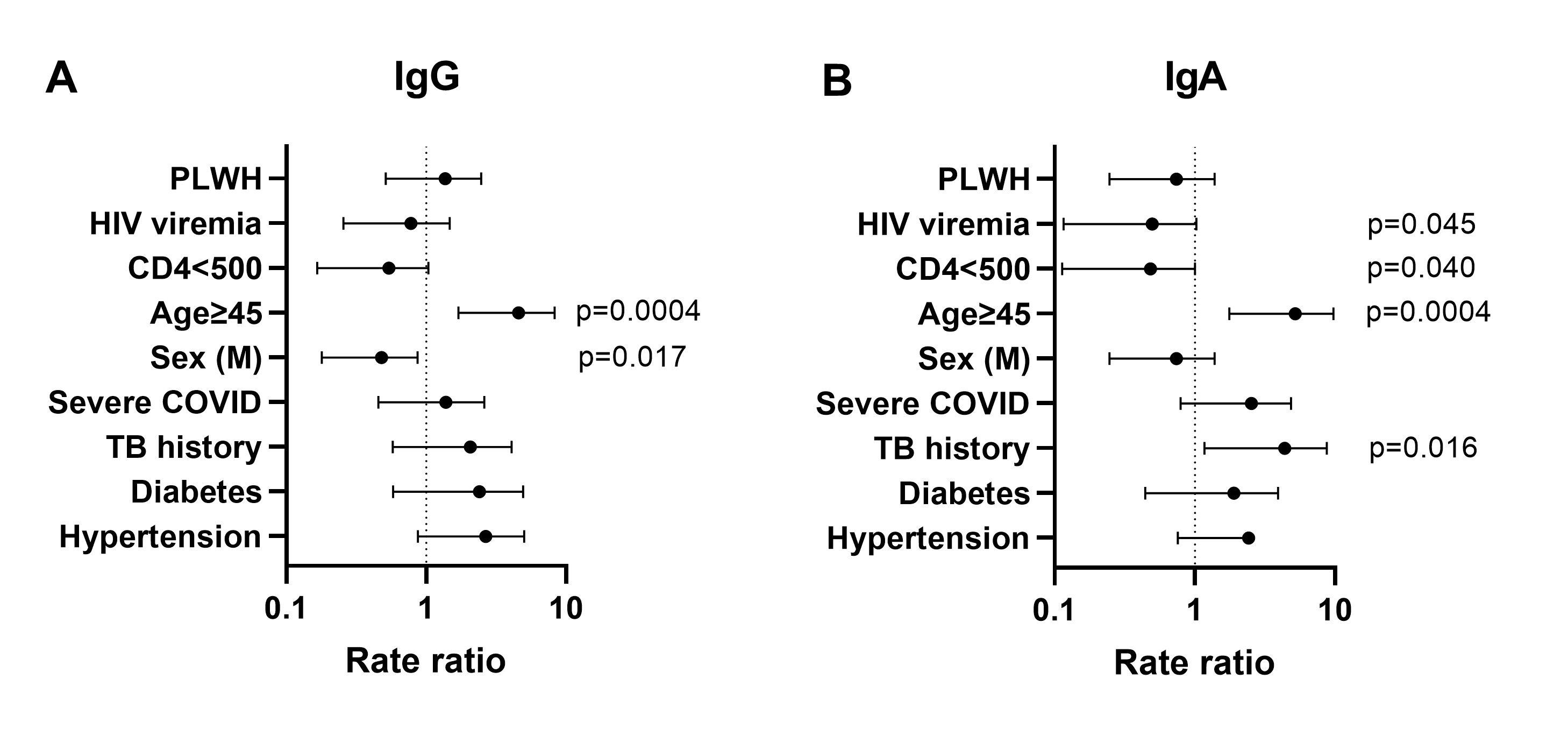

Supplement: jiac343_Supplementary_Data [file jiac343_supplementary_data.zip › FS1_Rate ratios IgG IgA_noCD8.tif]

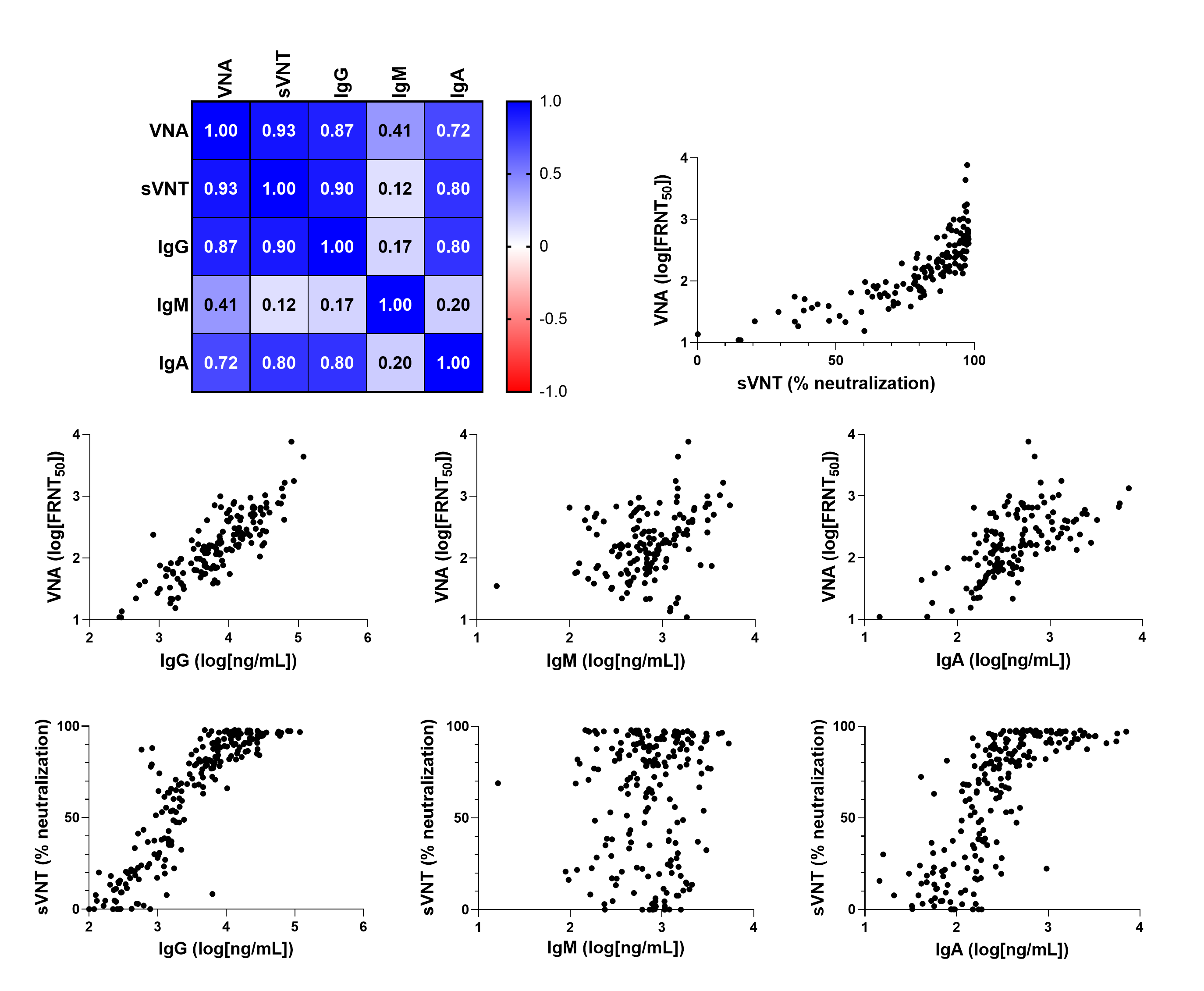

Supplement: jiac343_Supplementary_Data [file jiac343_supplementary_data.zip › FS2_Correlations all both waves_v3.tif]
